# Hyaluronic Acid Coated Acid-Sensitive Nanoparticles for Targeted Therapy of Adjuvant-Induced Arthritis in Rats

**DOI:** 10.3390/molecules24010146

**Published:** 2019-01-02

**Authors:** Changhui Yu, Xiangyu Li, Yufei Hou, Xiangxue Meng, Deli Wang, Jiaxin Liu, Fengying Sun, Youxin Li

**Affiliations:** School of Life Sciences, Jilin University, Changchun 130012, China; changhuiyujlu@126.com (C.Y.); lixiangyu17@mails.jlu.edu.cn (X.L.); houyf18@mail.jlu.edu.cn (Y.H.); mxx18@mails.jlu.edu.cn (X.M.); wangdl@jlu.edu.cn (D.W.); jxliu328@163.com (J.L.)

**Keywords:** rheumatoid arthritis, dexamethasone, hyaluronic acid, PCADK, acid-sensitive

## Abstract

Activated macrophages play a vital role in rheumatoid arthritis (RA) pathophysiology. CD44 is an overexpressed receptor on activated macrophages that is a potential target site for RA treatment. In this study, we prepared hyaluronic acid (HA) coated acid-sensitive polymeric nanoparticles (HAPNPs) composed of egg phosphatidylcholine, polyethylenimine, and poly (cyclohexane-1,4-diyl acetone dimethylene ketal) (PCADK) loaded with dexamethasone (Dex) for the treatment of RA. PCADK was used to form polymeric cores because of its acid-sensitivity. The HAPNPs were about 150 nm in size and had a zeta potential of −2.84 mV. The release rate of Dex from HAPNPs/Dex in vitro increased markedly when the pH decreased from 7.4 to 4.5, indicating that the HAPNPs were pH-sensitive. In a cellular uptake study, stronger fluorescence signals were observed in activated macrophages treated with HAPNPs, suggesting that HAPNPs could be effective nanodevices target to activated macrophages. In rats with adjuvant-induced arthritis, HAPNPs could inhibited the progression of RA. Taken together, these results suggest that the HAPNPs could be useful in RA therapy.

## 1. Introduction

Rheumatoid arthritis (RA) is a common chronic inflammatory autoimmune disease that causes bone destruction, hyperplasia of synovial membranes and cartilage damage [1,2,3]. Although the exact mechanism of RA remains unknown, abundant activated macrophages in the inflamed joints play a crucial role in the progression of the disease via the production of pro-inflammatory cytokines such as tumor necrosis factor alpha (TNF-α), interleukin-6 (IL-6) and IL-1β [4,5,6]. Therefore, inhibiting the secretion of pro-inflammatory cytokines from activated macrophages has been the primary target of RA therapy [7]. CD44 is an adhesion receptor that is often overexpressed on the surface of activated macrophages in RA patients [8,9]. Hyaluronic acid (HA), a natural polysaccharide, exhibits desirable biocompatibility and biodegradability and has been extensively applied in novel drug delivery systems [10,11,12,13,14,15]. It was selected as a targeting moiety for RA therapy due to its ability to bind CD44 [16].

The glucocorticoid (dexamethasone (Dex)) was widely applied in the treatment of various inflammatory diseases and has excellent anti-inflammatory efficacy [17]. However, its systemic use always causes severe side effects in normal tissues [18]. Targeted glucocorticoid delivery systems could be a promising potential solution to these side effects and have been extensively studied. 

Polyethylenimine (PEI) has been widely used in drug delivery system because of the proton sponge effect. It has been reported that PEI could combine with negatively charged materials due to the high positive charge of PEI [19]. Liposomes present a series of advantages as drug delivery applications because of the advantages of lipids (such as permeability, charge density, and steric hindrance) and there have been many studies reporting the potential applications of liposomes in RA treatment [15]. Egg phosphatidylcholine (egg PC), a kind of lipids, was also used in lipid-polymer hybrid nanoparticles [20].

Due to the process of extravasation through leaky vasculature and subsequent inflammatory cell-mediated sequestration (ELVIS) in inflamed joints, nano-sized drug delivery systems can passively accumulate in inflamed joints [2,21,22]. Nanoparticulate delivery systems such as liposomes, polymeric nanoparticles, gold nanoparticles are studied a lot [23,24]. The major problem associated with gold therapy is the high toxicity to rheumatoid arthritis patients which limited gold salts for the treatment of RA. However, liposomes have major drawbacks such as rapid clearance by the reticuloendothelial system, drug leakage, non-specific distribution and phospholipid oxidation in the liquid state which limited its application in drug delivery. Lipid–polymer hybrid nanoparticles possess favorable biocompatibility, high encapsulation efficiency, and excellent stability and have been studied in many fields in recent years [25,26]. In addition, lipid–polymer hybrid nanoparticles combine the advantages of liposomes and polymeric nanoparticles [27]. Although polymeric nanoparticles such as poly (d,l-lactic-co-glycolic acid) (PLGA) nanoparticles have been exploited in many diseases, their acidic degradation products, which themselves frequently cause inflammation limited its use in inflammatory diseases [28]. To enable the therapeutic agents to be released rapidly once the drug delivery systems reach the acidic micro-environment of the endosome or lysosome, pH-responsive drug delivery systems have been reported. Poly (cyclohexane-1,4-diyl acetone dimethylene ketal) (PCADK), a member of the polyketal family, has excellent properties of acid sensitivity, biocompatibility and biodegradability, and has been widely exploited in pH-responsive drug delivery systems [20,27,28,29,30,31].

In this study, we used PCADK, egg phosphatidylcholine (egg PC) and polyethylenimine (PEI) to prepare acid-sensitive polymeric nanoparticles (PNPs), which were then coated with HA. The acid-sensitivity of the HA coated nanoparticles (HAPNPs) was investigated using an in vitro release study and the properties of HAPNPs targeting activated macrophages were monitored using the RAW 264.7 cell line. The in vivo therapeutic effect of HAPNPs/Dex was investigated in rats with adjuvant-induced arthritis.

## 2. Results

### 2.1. Characterization of Nanoparticles

We previously reported that nanoparticles or microspheres based on a polyketal have pH sensitivity [20,32,33,34]. However, nanoparticles made with only PCADK have a large particle size. Egg PC and PEI were used to adjust the particle size and zeta potential of nanoparticles and prolong the duration of the blood cycle. The nanoparticles were prepared using an emulsion solvent evaporation method. The particle size, PDI and zeta potential of the different formulations were shown in Table 1. The particle size decreased significantly with the addition of egg PC from 0 mg to 8 mg. F5 was chosen for further investigation due to its desirable particle size (116.8 ± 1.41 nm). Zeta potential significantly increased as the amount of PEI increased from 0 mg to 1 mg. F11 was chosen for further optimization because it exhibited the highest zeta potential (25.83 ± 4.78 mV).

The effects of different amounts of HA in the PNP suspensions on the size distribution and zeta potential were also evaluated. As shown in Table 2, increasing HA from 0 μL to 500 μL effectively neutralized the superficial positive zeta potential. However, the size of the PNPs also increased significantly and high HA concentrations (≥600 μL) led to turbid nanosuspensions. Hence, we chose F15 as the optimized formulation for further investigation. The compositions and characteristics of F15 are shown in Table S1.

**Figure 1:**
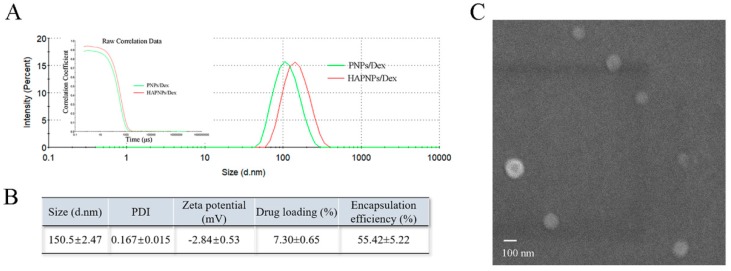
Characterization of acid-sensitive nanoparticles. (**A**) Dynamic light scattering size distribution of HAPNPs/Dex and PNPs/Dex. (**B**) Physicochemical properties of HAPNPs/Dex (mean ± SD, n = 3). (**C**) Scanning electron microscope image of HAPNPs/Dex (Scale bar, 100 nm).

Figure 1 shows that the HAPNPs/Dex exhibited an average diameter of 150.5 nm with a narrow size distribution. The DL% and EE% of Dex in HAPNPs/Dex were 7.30 ± 0.65% and 55.42 ± 5.22% respectively. The morphology of the HAPNPs/Dex was observed by SEM. As shown in Figure 1C, the HAPNPs were homogeneously distributed without aggregation.

### 2.2. Stability of the Nanoparticles

**Figure 2:**
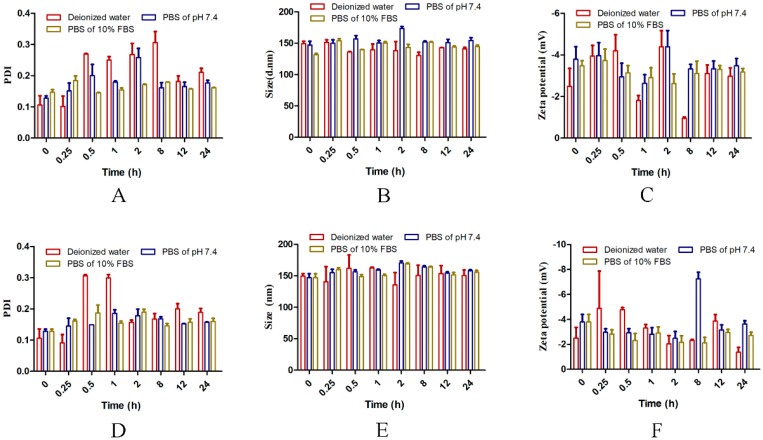
Stability of HAPNPs/Dex in deionized water, PBS and PBS containing 10% FBS. Size, PDI and zeta potential were measured at 4 °C (**A**–**C**) or at 37 °C (**D**–**F**). Data are presented as mean ± SD (n = 3).

The stability of the HAPNPs/Dex at 4 °C and 37 °C was investigated. Figure 2A–C shows that from 0 h to 24 h, particle size, PDI and zeta potential did not change significantly in water, PBS or PBS containing 10% FBS at 4 °C. Similarly, as shown in Figure 2D–F, particle size, PDI and zeta potential did not change significantly in different solvents from 0 h to 24 h at 37 °C. These results show that the nanoparticles would be stable in vivo for 24 h.

### 2.3. Hemolysis Assay

**Figure 3:**
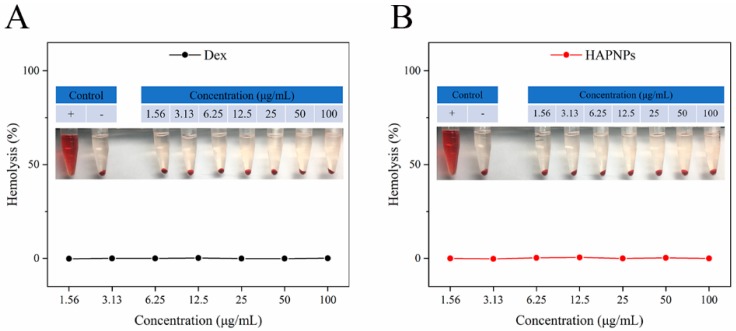
Hemolysis assay of free Dex solution (**A**) and HAPNPs/Dex (**B**).

Because the nanoparticles are intended for intravenous administration, biocompatibility is crucial for successful clinical application. A hemolysis assay was conducted to determine the biocompatibility of the nanoparticles. As shown in Figure 3A,B, no obvious hemolysis was observed; as the Dex concentration increased from 1.56 μg/mL to 100 μg/mL, the hemolysis rates of the Dex solution and HAPNPs/Dex were significantly lower than the allowed standard level of 5% [35]. These results indicate that our prepared nanoparticles exhibited excellent biocompatibility and could be used in an in vivo experiment via intravenous injection.

### 2.4. In Vitro Release of Dex from Nanoparticles

**Figure 4:**
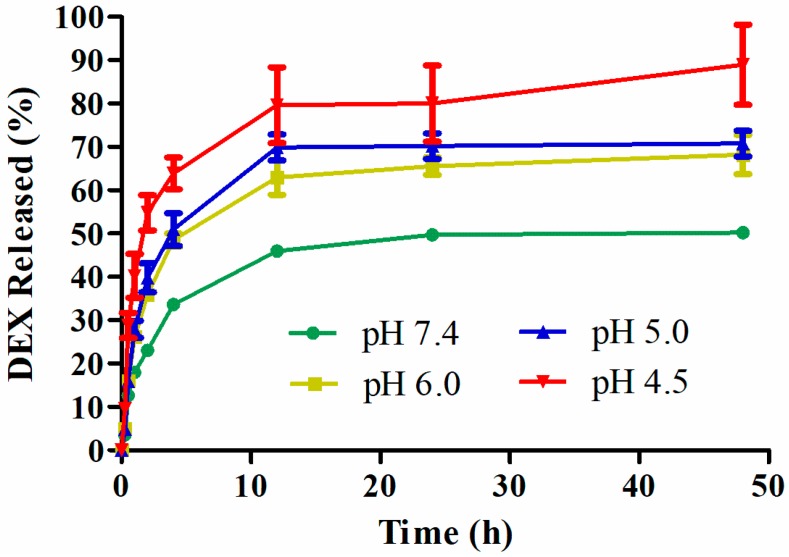
The pH-sensitive release profiles of Dex from HAPNPs/Dex at pH 7.4, 6.0, 5.0 and 4.5 at 37 °C. Data are presented as mean ± SD (n = 3).

The Dex release behavior from HAPNPs/Dex was monitored at different pH levels (7.4, 6.0, 5.0, and 4.5). As shown in Figure 4, as we expected, HAPNPs/Dex exhibited remarkable pH-responsive Dex release. There was burst release of Dex from HAPNPs/Dex at all pH levels within 15 min possibly caused by the drug being embedded in the surface or the outer layer of the HAPNPs. The release rates of Dex at pH 4.5, 5.0, and 6.0 were faster than at pH 7.4 from 0 h to 12 h. With the decrease in pH value, the amount of Dex released from the HAPNPs increased. Approximately 50%, 68%, 71% and 88% of Dex was released from HAPNPs/Dex at pH 7.4, 6.0, 5.0, and 4.5, respectively, indicating that the HAPNPs are pH sensitive, and Dex will release quickly from HAPNPs/Dex when it reaches an acidic environment.

### 2.5. Cytotoxicity Assays

**Figure 5:**
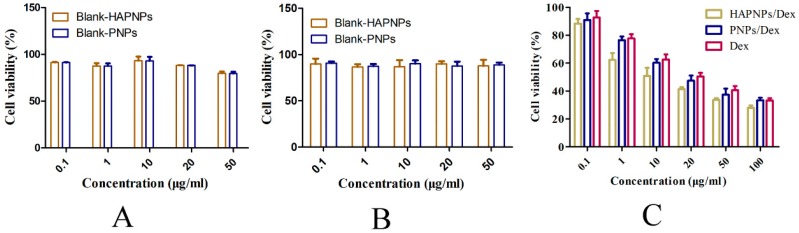
(**A**) Viability of RAW 264.7 cells after treatment with blank PNPs or HAPNPs at different concentrations for 24 h. (**B**) Viability of RAW 264.7 cells activated by LPS after treatment with blank PNPs or HAPNPs at different concentrations for 24 h.(**C**) Viability of RAW 264.7 cells activated by LPS after treatment with PNPs/Dex, HAPNPs/Dex or free Dex at different concentrations for 24 h. Results are presented as mean ± SD (n = 3).

The in vitro toxicity of Dex and HAPNPs/Dex was tested in cell culture. As shown in Figure 5A,B, the blank HAPNPs and blank PNPs were nontoxic to activated RAW 264.7 or RAW 264.7 cells at concentrations between 0.1 μg/mL and 50 μg/mL. Figure 5C shows that HAPNPs/Dex, PNPs/Dex and free Dex exhibited concentration-dependent cytotoxicity. The cytotoxicity of PNPs/Dex was similar to the cytotoxicity of free Dex. HAPNPs/Dex exhibited slightly higher cytotoxicity than PNPs/Dex and free Dex. Those results possibly because HAPNPs/Dex could be uptake by activated macrophages.

### 2.6. Cellular Uptake Study

**Figure 6:**
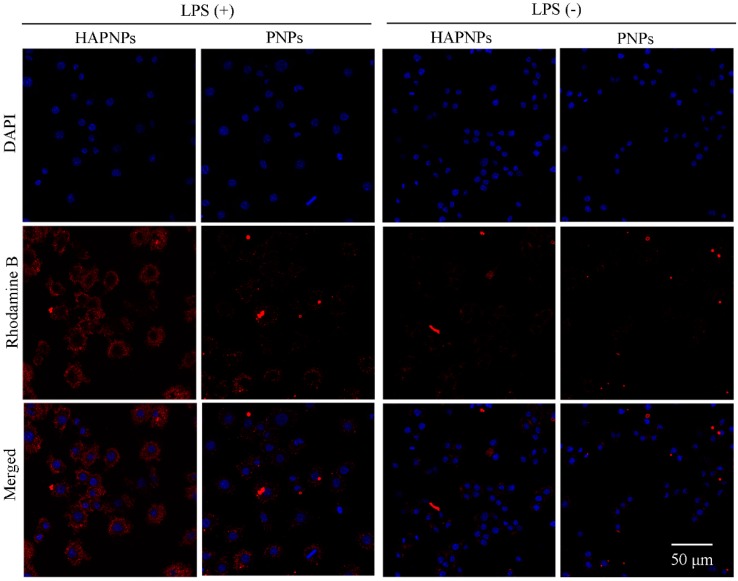
Confocal laser scanning microscope images of RAW 264.7 cells after activation by LPS (+) or not (−) incubated with rhodamine B-labeled PNPs and HAPNPs ((Scale bar, 50 μm)).

Activated macrophages play a crucial role in RA progression via the secretion of pro-inflammatory cytokines [7]. To investigate the cellular uptake behavior and targeting ability of HAPNPs toward activated macrophages, a CLSM examination was conducted. Nuclei were stained with DAPI, while rhodamine B was encapsulated into nanoparticles to assess their internalization by RAW 264.7 cells activated with LPS or not. As Figure 6 shows, after incubation with PNPs/rhodamine B or HAPNPs/rhodamine B, noticeable fluorescence intensity was not detected in non-activated RAW 264.7 cells. Compared to non-activated RAW 264.7 cells, strong fluorescence intensity of rhodamine B was observed in activated RAW 264.7 cells incubated with rhodamine B-labelled PNPs or rhodamine B-labeled HAPNPs, due to non-opsonic phagocytosis of activated macrophages [36]. Figure 6 also shows that the rhodamine B in activated macrophages treated with rhodamine B-labeled HAPNPs was markedly stronger than that in activated macrophages treated with rhodamine B-labeled PNPs. These results demonstrate that adding HA of HAPNPs promotes the internalization of PNPs by activated macrophages through CD44-mediated endocytosis.

### 2.7. In Vivo Therapeutic Efficacy of HAPNPs/Dex

**Figure 7:**
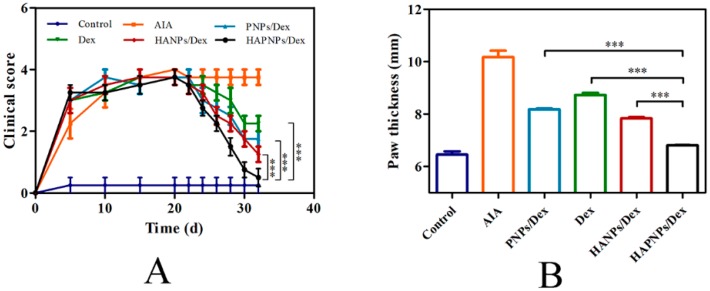
(**A**) Clinical score of rheumatoid arthritis as a function of days after induction. (**B**) Hind paw thickness of AIA rats on day 32 after induction. Results are presented as mean ± SD (n = 6, *** *p* < 0.001).

**Figure 8:**
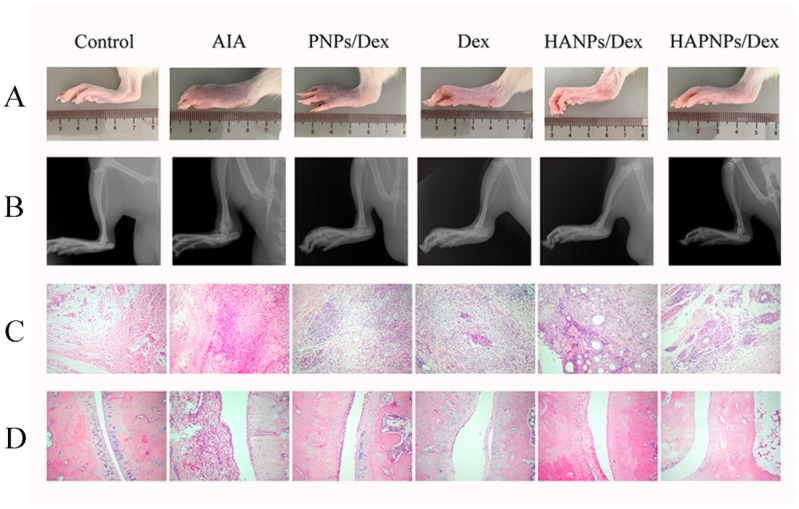
Therapeutic effects of HAPNPs/Dex, PNPs/Dex, HANPs/Dex and free Dex in AIA rats. (**A**) Photographs of AIA rat paws from the different groups. (**B**) Hind paws of AIA rats imaged by X-ray. (**C**) Periarticular soft tissues of hind paws histologically identified by H&E staining. (**D**) Ankle joints of hind paws in the different group histologically identified by H&E staining.

As expected, 20 days after induction, the AIA model was fully developed. The AIA rats were divided into five groups (n = 6), which were treated with saline, PNPs/Dex, free Dex solution, HANPs/Dex (the compositions and characteristics of HANPs/Dex were showed in Table S2.), or HAPNPs/Dex, respectively. The anti-inflammatory efficacy of the various formulations was evaluated by assessing clinical score, paw thickness and photographs of hind paws. As shown in Figure 7A, after the first administration on day 20, the clinical score decreased gradually in PNPs/Dex, free Dex solution, HANPs/Dex, or HAPNPs/Dex group. On day 32, the HAPNPs/Dex group had the lowest clinical score. Figure 7B shows that the paw thickness of the AIA rats treated with saline (10.49 mm) was significantly greater than that of normal rats (6.22 mm). And after administration with different nanoparticles loaded with Dex or free Dex solution, the rats’ paws were relatively thinner than the saline-treated AIA group. In comparison with free Dex solution, PNPs/Dex and HANPs/Dex, HAPNPs/Dex resulted in notable suppression of paw swelling. Consistent with the clinical score and paw thickness results, the photographs of paws demonstrated that HAPNPs/Dex relieved the swelling and erythema caused by inflammation (Figure 8A,B). The results confirm that HAPNPs/Dex had superior curative effect on AIA, which is likely due to their acid sensitivity and targeting of activated macrophages. 

### 2.8. Histological Analysis

The therapeutic effects of HAPNPs/Dex were further investigated by histological analysis. As shown in Figure 8C,D, when compared with normal rats, the AIA rats treated with saline exhibited cartilage damage, prominent inflammatory cell infiltration, and severe bone erosion. In the AIA rats treated with free Dex solution, these effects were still obvious. The AIA rats treated with PNPs/Dex and HAPNPs/Dex showed relatively reduced bone erosion and cartilage damage, and the prominent inflammatory cell infiltration in joints were also reduced. The ankle joints of the AIA rats treated with HAPNPs/Dex exhibited the greatest reductions in cartilage damage, inflammatory cell infiltration, and bone damage. These results are consistent with the in vivo therapeutic efficacy and cytokine assay results. Bone erosion and cartilage damage caused by excessive secretion of pro-inflammatory cytokines are common symptoms of RA. Based on the results of the cytokine assay, the excellent therapeutic effects of HAPNPs/Dex on tissue damage were probably a result of inhibiting the secretion of pro-inflammatory cytokines. 

### 2.9. Cytokine Assay

**Figure 9:**
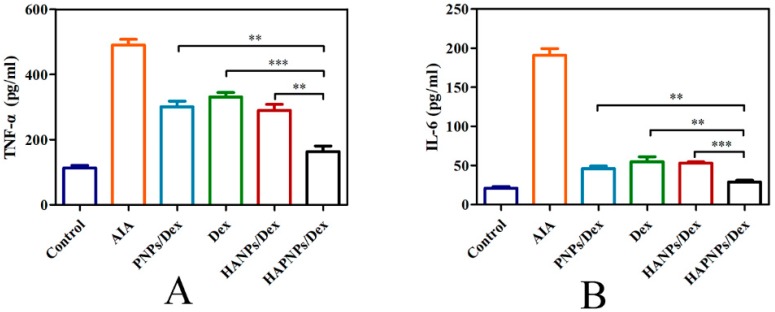
Level of TNF-α (**A**) and level of IL-6 (**B**). Results are presented as mean ± SD (n = 6, ** *p* < 0.01, *** *p* < 0.001).

It is well known that pro-inflammatory cytokines perform important functions in the pathogenesis of RA. In this study, on day 32 after AIA induction, ELISA kits were used to determine the serum levels of TNF-α and IL-6. As shown in Figure 9A,B, the serum levels of TNF-α and IL-6 in AIA rats treated with saline were significantly increased compared with normal rats, at 491 pg/mL and 191 pg/mL, respectively. Interestingly, the levels of TNF-α and IL-6 in the AIA rats treated with HAPNPs/Dex were 150 pg/mL and 20 pg/mL, respectively, approaching similar levels to those of the normal rats. In RA, activated macrophages play a crucial role in inducing and aggravating inflammation through the secretion of pro-inflammatory cytokines. Therefore, the therapeutic effect of HAPNPs/Dex on AIA rats could be attributed to the targeting characteristic of CD44 on activated macrophages and superior cytotoxicity.

## 3. Discussion

Nanoparticles as a new drug delivery system is widely used in cancer therapy and inflammation therapy over the past decades. Here we used biocompatible, biodegradable and acid sensitive polymer PCADK to encapsulate Dex in nanoparticles, and then coated with HA for RA therapy [30]. The results showed that this formation could suppress paw swelling and erythema, and prominent inflammatory cell infiltration, bone damage and cartilage damage were also reduced in the ankle joints of AIA rats. 

Particle size is important for intravenous applications. The diameter of the smallest capillaries is around 2–3 μm [37]. Thus the capillaries would not be clogged when particles are less than 1.5 μm in diameter. Generally, particles over 200 nm in diameter would be eliminated by spleen filtration system and less than 10 nm easily penetrate the kidney’s filtering systems [38]. On the other hand, due to the process of extravasation through leaky vasculature and subsequent inflammatory cell-mediated sequestration (ELVIS) which is similar to enhanced permeability and retention (EPR) effect in inflamed joints [2,39]. Nanoparticles would more easily bioaccumulate in inflamed joints. In this experiment, the HAPNPs/Dex exhibited an average diameter of 150.5 nm (Table 2) which is indicated that our nanoparticles are suitable for intravenous administration and could bioaccumulate in inflamed joints.

Activated macrophages have great impact on the progression of inflammatory diseases, such as RA, osteoarthritis, lupus and cancer [20]. Plenty of activated macrophages would assemble in RA joints, which could secrete some pro-inflammatory cytokines such as TNF-α and IL-1β which could accelerate the progression of rheumatoid arthritis. Pro-inflammatory cytokines played an important role in the progression of RA. We measured the levels of TNF-a and IL-1β on day 32 after AIA induction. The serum levels of TNF-α and IL-6 in AIA rats treated with HAPNPs/Dex were significantly decreased compared with AIA rats with saline (Figure 9A,B). Besides, activated macrophages would excessively express CD44 which could specifically bind HA [16]. Therefore, the activated macrophages are an important issue for active targeting therapy. Our experiments showed that HAPNPs could uptake into LPS-activated macrophages. Uptake by LPS-activated macrophages was significantly higher than by normal macrophages. The uptake of HAPNPs into activated macrophages was significantly higher than uptake of PNPs (Figure 6). And in cytotoxicity assays studies, the cell viability of activated macrophages treated HAPNPs/Dex was lower than that treated with PNPs/Dex and free Dex (Figure 5). All the results indicated our nanoparticles could targeted activated macrophages overexpressed CD44. 

Stability and biocompatibility are also important for the storage and application of nanoparticles [27]. The stability of the HAPNPs/Dex in water, PBS or PBS containing 10% FBS at 4 °C and 37°C was investigated. Figure 2A–F shows that from 0 h to 24 h, particle size, PDI and zeta potential did not change significantly. These results show that the nanoparticles would be stable in vivo for 24 h. A hemolysis assay was conducted to determine the biocompatibility of the nanoparticles. As shown in Figure 3A,B, no obvious hemolysis was observed. These results indicate that our prepared nanoparticles exhibited excellent biocompatibility and safety for intravenous administration. 

It is reported that the pH of blood is about 7.4, lysosomes were 4–5, endosomes were 5–6 and inflammatory tissues were about 6.5 [20,40]. And pH-sensitive nanoparticles were widely used in RA therapy because of the pH profile in inflammatory joints is lower than that of normal tissues [41,42]. In vitro release studies, Dex could release quickly from HAPNPs/Dex at pH 4.5 (Figure 4). Thus, Dex would rapidly release when the acid-sensitive nanoparticles were taken up into endosome. Additionally, HA could prolong long retention in circulation and increase the accumulation of nanoparticles in inflamed joints. Those results may explain why HAPNPs/Dex had a better therapeutic efficacy than free Dex and PNPs/Dex. Poly (d,l-lactic-co-glycolic acid) (PLGA) has been utilized in many drug delivery systems on the market due to its favorable biocompatibility and biodegradability. In recent decades, PLGA nanoparticles have been exploited in many diseases. However, PLGA is not acid sensitive compared with PCADK, and its acid degradation product lactic acid and glycolic acid were unfavorable on inflammatory disease [28]. This may explain why HAPNPs/Dex had a better therapeutic efficacy than HANPs/Dex. Furthermore, PCADK would degrade to acetone and 1,4-cyclohexanedimethanol which were neutral and biocompatible. It makes our nanoparticles have great prospects in RA inflammatory disease.

The anti-inflammatory efficacy of glucocorticoids as anti-inflammatory agents is previously established [43]. The anti-inflammatory efficacy of the various formulations was evaluated by assessing clinical score, paw thickness, photographs of hind paws and histological analysis. As shown in Figure 7A,B and Figure 8, on day 32, the HAPNPs/Dex group had the lowest clinical score. And HAPNPs/Dex resulted in notable suppression of paw swelling. Consistent with the clinical score and paw thickness results, the photographs of paws demonstrated that HAPNPs/Dex relieved the swelling and erythema caused by inflammation. The ankle joints of the AIA rats treated with HAPNPs/Dex exhibited the greatest reductions in cartilage damage, inflammatory cell infiltration, and bone damage. The results confirm that HAPNPs/Dex had superior curative effect on AIA, which is likely due to their acid sensitivity and targeting of activated macrophages.

## 4. Materials and Methods

### 4.1. Materials

Dex was purchased from Shanghai Yuanye Bio-Technology Co., Ltd. (Shanghai, China). Egg PC was purchased from Seebio Biotech (Shanghai) Co., Ltd. (Shanghai, China). Sodium hyaluronate (HA, Mw < 10 K) was purchased from Lifecore Biomedical Co., Ltd (Chaska, MN, USA). PEI (Mw 25 kDa, branched), polyvinyl alcohol (PVA), dichloromethane, and acetone were purchased from Sigma–Aldrich (St. Louis, MO, USA). Fetal bovine serum (FBS) and RAW 264.7 cells were obtained from Wuhan Procell Biological Technology Co., Ltd (Wuhan, China). 4′,6-Diamidino-2-phenylindole dihydrochloride (DAPI) was obtained from Beyotime Institute of Biotechnology (Shanghai, China). Rhodamine B was obtained from Sinopharm Chemical Reagent Co., Ltd. (Beijing, China). Complete Freund’s adjuvant (CFA) was obtained from Chondrex (Redmond, WA, USA). Rat plasma tumor necrosis factor alpha (TNF-α) ELISA kits and interleukin (IL)-6 ELISA kits were obtained from Elabscience Biotechnology Co., Ltd (Wuhan, China). All other chemical reagents and solvents used were of analytical grade.

### 4.2. Preparation of Nanoparticles

Synthesis of PCADK was completed according to methods described previously [28]. To prepare positively charged lipid-polymer hybrid nanoparticles, we dissolved PCADK, egg PC and PEI in 700 μL of dichloromethane. Then 4 mg of Dex was dissolved in 350 μL of acetone and added to the organic phase. The mixture was then added to 2 mL of 2% PVA within 30 s under sonication at 300 W in an ice bath and then sonicated for 2 min under the same condition. The obtained emulsion was added to 35 mL aqueous solution of PVA (0.5%, *w/v*) and evaporated for 4 h at room temperature. Then 0.1% HA was added and the mixture was stirred overnight [44]. The nanoparticles were collected by centrifugation (Allegra 64R, Beckman Coulter, Brea, CA, USA) conducted three times (20000 rpm, 30 min, 4 °C). 

### 4.3. Characterization of Nanoparticles

The particle size, polydispersity index (PDI) and zeta potential of the nanoparticles were measured using dynamic light scattering (DLS) (Zetasizer Nano ZS90, Malvern, Worcestershire, UK). And then we diluted the nanoparticles to detecting concentration (1 mg/mL) with deionized water and characterized at 25 °C.

The morphology of the nanoparticles was determined with a scanning electron microscope (SEM) (JXA-840, JEOL, Tokyo, Japan) at 3 kV accelerating voltage. Briefly, 1 mg/mL of nanoparticles were dropped on a silicon wafer and left at room temperature to evaporate water prior to SEM observation. 

The drug loading (DL) and encapsulation efficiency (EE) of Dex in HAPNPs/Dex were determined as follows. First, we dissolved 2 mg of the HAPNPs/Dex in 500 μL of dichloromethane and then added 4.5 mL of mobile phase. The mixture was then centrifuged at 10,000 rpm for 10 min, and the supernatant was collected and analyzed by a Waters high-performance liquid chromatography (HPLC) system equipped with a 1525 binary LC pump and a 2487 UV vis dual absorbance detector (Waters Corp., Milford, MA, USA) set at 240 nm. An Agilent Extend C18 column (5 µm, 4.6 × 250 mm, Agilent Technologies, Inc., Santa Clara, CA, USA) was used for drug separation with a mobile phase of acetonitrile: water 60:40 (*v/v*). The flow rate and the injection volume were set at 1 mL/min and 20 μL, respectively. The temperature was 30 °C. The actual DL and EE of the nanoparticles were calculated as follows.

$$\mathrm{DL}\;\left(\%\right)=\frac{\mathrm{amount}\;\mathrm{of}\;\mathrm{drug}\;\mathrm{encapsulated}\;\mathrm{in}\;\mathrm{HPNPs}/\mathrm{Dex}}{\mathrm{amount}\;\mathrm{of}\;\mathrm{HPNPs}/\mathrm{Dex}}\times 100\%,$$

$$\mathrm{EE}\;\left(\%\right)=\frac{\mathrm{actual}\;\mathrm{drug}\;\mathrm{loading}\;}{\mathrm{theoretical}\;\mathrm{drug}\;\mathrm{loading}}\times 100\%,$$

where DL and EE represent drug loading and the entrapment efficiency, respectively.

### 4.4. Stability of Nanoparticles

HAPNPs/Dex were suspended in deionized water, phosphate buffered saline (PBS) of pH 7.4, or PBS of pH 7.4 with 10% FBS and then incubated at 37 °C and 4 °C. The sizes and polydispersity indexes of the HAPNPs/Dex nanoparticles were measured using DLS at predetermined time points to evaluate their stability.

### 4.5. Hemolysis Assay

As favorable biocompatibility is crucial if HAPNPs/Dex are to be applied through intravenous administration, a hemolysis assay was conducted. First, whole blood was collected from the rat orbit. To obtain red blood cells (RBCs), the blood sample was centrifuged at 5000 rpm for 10 min at 4 °C and then washed three times with normal saline. HAPNPs/Dex or free Dex solutions of predetermined concentrations were added to the 2% RBC suspension and then incubated at 37 °C for 3 h. Triton X-100 were used as positive control and the normal saline were used as the negative control. The collected supernatants were transferred to 96-well plates and determined at 540 nm (DNM-9602, PERLONG, Beijing, China). The hemolysis rate was defined using the following equation:

$$\mathrm{Hemolysis}\;\mathrm{rate}=\frac{A_{\mathrm{Sample}}-A_{\mathrm{Negative}\;\mathrm{control}}}{A_{\mathrm{Positive}\;\mathrm{control}}-A_{\mathrm{Negative}\;\mathrm{control}}}$$

where A_sample_, A_positive control_ and A_negative control_ represent the absorbance of the sample, positive control and negative control, respectively.

### 4.6. In Vitro Release of Dex from Nanoparticles

The release of Dex from nanoparticles were investigated by incubation of nanoparticles in PBS at pH 7.4, 6.0, 5.0 and 4.5. Briefly, 10 mg of nanoparticles were suspended in 2 mL of PBS at different pH and introduced into dialysis bags (MW cut-off: 100,000 Da), which were then immersed in 10 mL of release medium. At pre-specified time intervals, 3 mL of supernatant was collected and replaced with an equivalent amount of PBS and the Dex concentrations were measured by HPLC. 

### 4.7. Cellular Uptake Study

The cellular uptake of the nanoparticles was evaluated using RAW 264.7 cells. RAW 264.7 cells were incubated at 37 °C in a 5% CO_2_ in Dulbecco’s modified Eagle’s medium (DMEM) with 10% FBS. Briefly, 2 × 10^5^ cells/well were seeded in a 12-well plate, with 1 μg/mL lipopolysaccharide added to activate the macrophages. The cells were incubated for 48 h to grow and expand, and the culture medium was replaced with complete DMEM containing rhodamine B-labeled nanoparticles. After 2 h, the cells were washed three times with serum-free DMEM medium. Subsequently, the cells were fixed with 4% (*v/v*) paraformaldehyde for 15 min and the nuclei were counterstained with DAPI for 3 min. Finally, the cells were washed three times with ice-cold PBS and the cellular uptake of various nanoparticles was confirmed using a confocal laser scanning microscope (CLSM) on an LSM710 from Carl Zeiss Meditec (Jena, Germany).

### 4.8. Cytotoxicity Study

The cytotoxicity of the nanoparticles and free Dex solution were investigated. 1 × 10^4^ cells/well of RAW 264.7 cells were seeded in 96-well plates and activated with 1 μg/mL of LPS. After incubation for 48 h, the medium was discarded and replaced with fresh culture medium containing nanoparticles or free Dex solution at different concentrations (0.1 μg/mL, 1 μg/mL, 10 μg/mL, 20 μg/mL, 50 μg/mL and 100 μg/mL). After incubation for 24 h, 20 μL of MTT solution (5 mg/mL in PBS) was added to each well and further incubated for 4 h. Subsequently, the MTT-containing medium was removed and the formazan precipitate was dissolved using 150 μL of sterile dimethyl sulfoxide. After incubation for 10 min at 37 °C, the plates were carefully shaken for 1 min and the UV absorbance at 490 nm was measured using a microplate reader (DNM-9602, PERLONG, China). The cell viability was calculated according to the following equation:

$$\mathrm{Cell}\;\mathrm{viability}=\frac{A_{\mathrm{sample}}-A_{\mathrm{blank}}}{A_{\mathrm{negative}\;\mathrm{control}}-A_{\mathrm{blank}}},$$

### 4.9. In Vivo Therapeutic Efficacy of HAPNPs/Dex 

An adjuvant-induced arthritis (AIA) model was established using the method as described previously [45]. Briefly, 36 male Sprague Dawley rats weighting 160–180 g were injected with 50 μL of CFA (10 mg/mL) subcutaneously at the left footpad. On day 20 after AIA model establishment, the rats were randomly divided into six groups (n = 6): (a) normal (healthy rats without arthritis treated with saline), (b) saline (AIA rats treated with saline), (c) free Dex (AIA rats treated with free Dex), (d) polylactic-co- glycolic acid (PLGA) nanoparticles (HANPs) (AIA rats treated with HANPs/Dex), (e) PNPs/Dex (AIA rats treated with PNPs/Dex), (f) HAPNPs/Dex (AIA rats treated with HAPNPs/Dex). The rats were treated with the different formulations every two days starting on day 20 after the AIA model was induced. The dose of Dex was 5 mg/kg. The arthritis severity of the rats was scored with the following system [46]: 0 = no erythema or swelling, 1 = slight swelling and confined erythema, 2 = slight swelling and extended erythema, 3 = moderate swelling and extended erythema, 4 = severe swelling and widespread erythema. In addition, the thickness of the hind paws was measured using calipers.

All of the animal experiments were performed in accordance with legal and institutional guidelines. The procedures were approved by the Ethical Committee for Care and Use of Laboratory Animals at Jilin University (201804001).

### 4.10. Cytokine Assay

On day 32 after model induction, blood was collected and then centrifuged to separate the serum. The serum concentrations of TNF-α and IL-6 were determined using ELISA kits (Elabscience Biotechnology, Wuhan, China) according to the manufacturer’s instructions.

### 4.11. Histological Analysis

After the blood samples were collected, the rats were euthanized for histological analysis. Briefly, the ankle joints of rats were dissected then fixed in 4% (*w/v*) buffered paraformaldehyde for 24 h, and decalcified in 10% (*w/v*) EDTA at 37 °C for 4 weeks. Then the tissues were embedded in paraffin and sliced into sections with 3 mm thick. Finally, the sections were stained with hematoxylin and eosin (H&E) and visualized using an Olympus confocal microscope.

### 4.12. Statistical Analysis

The results were expressed as mean ± standard deviation (SD). Origin 8.0 was used to compare mean values. Group statistical significance was measured using one-way ANOVA. Statistical significance was assigned as * *p* < 0.05, ** *p* < 0.01 and *** *p* < 0.001. A *p* value <0.05 was considered statistically significant.

## 5. Conclusions

Hyaluronic acid coated acid-sensitive nanoparticles (HAPNPs) for the targeted therapy of RA were developed. The HAPNPs were of appropriate size and zeta potential for use as a systemic medication by intravenous injection. The results of in vitro release experiments proved that the HAPNPs are pH sensitive. The cellular uptake and cytotoxicity studies showed that they could target activated macrophages because of overexpressed CD44. Most importantly, in vivo therapeutic efficacy studies showed that prominent inflammatory cell infiltration, bone damage and cartilage damage were reduced in the ankle joints of AIA rats treated with HAPNPs/Dex. Overall, these results suggest that HAPNPs can be used as a novel treatment mode for inflammatory diseases.

## Figures and Tables

**Table 1:** Compositions and characteristics of polymeric nanoparticles (PNPs). (n = 3).

Batch	PCADK (mg)	Egg PC (mg)	PEI (mg)	Size (d.nm)	PDI	Zeta Potential (mV)
F1	20	-	-	177.8 ± 6.90	0.116 ± 0.012	−0.79 ± 2.44
F2		2		163.8 ± 5.0	0.153 ± 0.025	−3.77 ± 2.31
F3		4		148.8 ± 8.89	0.202 ± 0.060	−8.61 ± 1.64
F4		6		123.6 ± 4.79	0.209 ± 0.052	−14.35 ± 2.39
F5		8		116.8 ± 1.41	0.336 ± 0.042	−16.00 ± 0.17
F6		10		115.7 ± 1.42	0.254 ± 0.112	−17.10 ± 3.81
F7		8	0.2	118.6 ± 5.91	0.158 ± 0.010	−9.07 ± 4.25
F8			0.4	118.3 ± 3.55	0.159 ± 0.010	8.33 ± 4.47
F9			0.6	119.1 ± 3.55	0.164 ± 0.057	11.67 ± 3.31
F10			0.8	121.2 ± 5.61	0.162 ± 0.011	16.20 ± 3.19
F11			1	121.6 ± 2.21	0.167 ± 0.013	25.83 ± 4.78
F12			1.2	122.6 ± 3.27	0.169 ± 0.016	24.18 ± 5.51

Data are expressed as mean ± standard deviation (SD).

**Table 2:** Optimization of hyaluronic acid (HA) amounts for coating PNPs. (n = 3).

Batch	HA (μL)	Size (d.nm)	PDI	Zeta Potential (mV)
F13	0	121.6 ± 2.21	0.167 ± 0.013	25.83 ± 4.78
F14	200	129.3 ± 2.89	0.260 ± 0.010	17.50 ± 0.57
F15	500	150.5 ± 2.47	0.167 ± 0.015	−2.84 ± 0.53
F16	600	509.5 ± 12.42	0.33 ± 0.014	−11.75 ± 1.13

Data are expressed as mean ± SD.

## Supplementary Materials

Supplementary Materials are available online. Figure S1: The XPRD spectra of PCADK, Table S1: Compositions and characteristics of HAPNPs (n = 3), Table S2: Compositions and characteristics of HANPs (n = 3).

## Abbreviations

RA	rheumatoid arthritis
HA	hyaluronic acid
HAPNPs	hyaluronic acid coated acid-sensitive polymeric nanoparticles
egg PC	egg phosphatidylcholine
PEI	polyethylenimine
PCADK	poly (cyclohexane-1,4-diyl acetone dimethylene ketal)
Dex	dexamethasone
TNF-α	tumor necrosis factor alpha
IL-6	interleukin-6
IL-1β	interleukin-1β
ELVIS	extravasation through leaky vasculature and subsequent inflammatory cell-mediated sequestration
PNPs	polymeric nanoparticles
PDI	polydispersity index
DL	drug loading
EE	encapsulation efficiency
SEM	scanning electron microscope
PBS	phosphate buffered saline
FBS	Fetal bovine serum
CLSM	confocal laser scanning microscope
DAPI	Diamidino-2-phenylindole dihydrochloride
LPS	lipopolysaccharide
AIA	adjuvant-induced arthritis
ELISA	enzyme-linked immunosorbent assay
H&E	hematoxylin and eosin
PVA	polyvinyl alcohol
CFA	Complete Freund’s adjuvant
DLS	dynamic light scattering
HPLC	high-performance liquid chromatography
RBCs	red blood cells
DMEM	Dulbecco’s modified Eagle’s medium
SD	standard deviation

## References

[1] O’Dell J.R. (2004). Drug therapy—Therapeutic strategies for rheumatoid arthritis. N. Engl. J. Med..

[2] Wang Q., Jiang J.Y., Chen W.F., Jiang H., Zhang Z.R., Sun X. (2016). Targeted delivery of low-dose dexamethasone using PCL-PEG micelles for effective treatment of rheumatoid arthritis. J. Control. Release.

[3] Firestein G.S. (2003). Evolving concepts of rheumatoid arthritis. Nature.

[4] Choy E.H.S., Panayi G.S. (2001). Mechanisms of disease: Cytokine pathways and joint inflammation in rheumatoid arthritis. N. Engl. J. Med..

[5] Shin J.M., Kim S.H., Thambi T., You D.G., Jeon J., Lee J.O., Chung B.Y., Jo D.G., Park J.H. (2014). A hyaluronic acid-methotrexate conjugate for targeted therapy of rheumatoid arthritis. Chem. Commun..

[6] Kim Y.J., Chae S.Y., Jin C.H., Sivasubramanian M., Son S., Choi K.Y., Jo D.G., Kim K., Kwon I.C., Lee K.C. (2010). Ionic complex systems based on hyaluronic acid and PEGylated TNF-related apoptosis-inducing ligand for treatment of rheumatoid arthritis. Biomaterials.

[7] Yang M.D., Ding J.X., Zhang Y., Chang F., Wang J.C., Gao Z.L., Zhuang X.L., Chen X.S. (2016). Activated macrophage-targeted dextran-methotrexate/folate conjugate prevents deterioration of collagen-induced arthritis in mice. J. Mater. Chem. B.

[8] Yang M., Feng X., Ding J., Chang F., Chen X. (2017). Nanotherapeutics relieve rheumatoid arthritis. J. Control. Release.

[9] Pure E., Cuff C.A. (2001). A crucial role for CD44 in inflammation. Trends Mol. Med..

[10] Choi K.Y., Chung H., Min K.H., Yoon H.Y., Kim K., Park J.H., Kwon I.C., Jeong S.Y. (2010). Self-assembled hyaluronic acid nanoparticles for active tumor targeting. Biomaterials.

[11] Naor D., Nedvetzki S. (2003). CD44 in rheumatoid arthritis. Arthritis Res. Ther..

[12] Heo R., Park J.S., Jang H.J., Kim S.H., Shin J.M., Suh Y.D., Jeong J.H., Jo D.G., Park J.H. (2014). Hyaluronan nanoparticles bearing gamma-secretase inhibitor: In vivo therapeutic effects on rheumatoid arthritis. J. Control. Release.

[13] Choi K.Y., Min K.H., Na J.H., Choi K., Kim K., Park J.H., Kwon I.C., Jeong S.Y. (2009). Self-assembled hyaluronic acid nanoparticles as a potential drug carrier for cancer therapy: synthesis, characterization, and in vivo biodistribution. J. Mater. Chem..

[14] Lapcik L., Lapcik L., De Smedt S., Demeester J., Chabrecek P. (1998). Hyaluronan: Preparation, Structure, Properties, and Applications. Chem. Rev..

[15] Oliveira I.M., Goncalves C., Reis R.L., Oliveira J.M. (2018). Engineering nanoparticles for targeting rheumatoid arthritis: Past, present, and future trends. Nano Res..

[16] Haynes B.F., Hale L.P., Patton K.L., Martin M.E., McCallum R.M. (1991). Measurement of an adhesion molecule as an indicator of inflammatory disease activity. Up-regulation of the receptor for hyaluronate (CD44) in rheumatoid arthritis. Arthritis Rheum..

[17] Baschant U., Lane N.E., Tuckermann J. (2012). The multiple facets of glucocorticoid action in rheumatoid arthritis. Nat. Rev. Rheumatol..

[18] Krasselt M., Baerwald C. (2014). The current relevance and use of prednisone in rheumatoid arthritis. Expert Rev. Clin. Immunol..

[19] Yu K.T., Zhao J.L., Zhang Z.K., Gao Y., Zhou Y.L., Teng L.S., Li Y.X. (2016). Enhanced delivery of Paclitaxel using electrostatically-conjugated Herceptin-bearing PEI/PLGA nanoparticles against HER-positive breast cancer cells. Int. J. Pharm..

[20] Zhao J.L., Zhao M.H., Yu C.H., Zhang X.Y., Liu J.X., Cheng X.W., Lee R.J., Sun F.Y., Teng L.S., Li Y.X. (2017). Multifunctional folate receptor-targeting and pH-responsive nanocarriers loaded with methotrexate for treatment of rheumatoid arthritis. Int. J. Nanomed..

[21] Wang D., Goldring S.R. (2011). The bone, the joints and the Balm of Gilead. Mol. Pharm..

[22] Adamo V., Ricciardi G., Schifano S., Russo A., Gebbia V., Blasi L., Giuffrida D., Scandurra G., Savarino A., Butera A. (2017). Safety and efficacy of the treatment with Nab-paclitaxel in mEtastatic bREast cancer In elDerly patiEnts: NEREIDE study. Ann. Oncol..

[23] Lee H., Lee M.Y., Bhang S.H., Kim B.S., Kim Y.S., Ju J.H., Kim K.S., Hahn S.K. (2014). Hyaluronate-Gold Nanoparticle/Tocilizumab Complex for the Treatment of Rheumatoid Arthritis. ACS Nano.

[24] Desai P.R., Marepally S., Patel A.R., Voshavar C., Chaudhuri A., Singh M. (2013). Topical delivery of anti-TNF alpha siRNA and capsaicin via novel lipid-polymer hybrid nanoparticles efficiently inhibits skin inflammation in vivo. J. Control. Release.

[25] Yang X., Grailer J.J., Rowland I.J., Javadi A., Hurley S.A., Matson V.Z., Steeber D.A., Gong S. (2010). Multifunctional stable and pH-responsive polymer vesicles formed by heterofunctional triblock copolymer for targeted anticancer drug delivery and ultrasensitive MR imaging. ACS Nano.

[26] Koch A.E. (2003). Angiogenesis as a target in rheumatoid arthritis. Ann. Rheum. Dis..

[27] Zhao J., Zhang X., Sun X., Zhao M., Yu C., Lee R.J., Sun F., Zhou Y., Li Y., Teng L. (2018). Dual-functional lipid polymeric hybrid pH-responsive nanoparticles decorated with cell penetrating peptide and folate for therapy against rheumatoid arthritis. Eur. J. Pharm. Biopharm..

[28] Yang S.C., Bhide M., Crispe I.N., Pierce R.H., Murthy N. (2008). Polyketal copolymers: A new acid-sensitive delivery vehicle for treating acute inflammatory diseases. Bioconjugate Chem..

[29] Fiore V.F., Lofton M.C., Roser-Page S., Yang S.C., Roman J., Murthy N., Barker T.H. (2010). Polyketal microparticles for therapeutic delivery to the lung. Biomaterials.

[30] Lee S., Yang S.C., Heffernan M.J., Taylor W.R., Murthy N. (2007). Polyketal microparticles: A new delivery vehicle for superoxide dismutase. Bioconjugate Chem..

[31] Heffernan M.J., Murthy N. (2005). Polyketal nanoparticles: A new pH-sensitive biodegradable drug delivery vehicle. Bioconjugate Chem..

[32] Wang C.H., Yu C.H., Yu K.T., Teng L.S., Liu J.X., Wang X.S., Sun F.Y., Li Y.X. (2016). Improving Protein Stability and Controlling Protein Release by Adding Poly (Cyclohexane-1, 4-diyl Acetone Dimethylene Ketal) to PLGA Microspheres. Curr. Drug Deliv..

[33] Wang C., Yu C., Liu J., Sun F., Teng L., Li Y. (2015). Stabilization of Human Immunoglobulin G Encapsulated within Biodegradable Poly (Cyclohexane-1, 4-diyl Acetone Dimethylene Ketal) (PCADK)/ Poly (Lactic-co-Glycolic Acid) (PLGA) Blend Microspheres. Protein Peptide Lett..

[34] Wang C.H., Yu C.H., Liu J.X., Teng L.S., Sun F.Y., Li Y.X. (2015). Preparation and in vivo evaluation of PCADK/PLGA microspheres for improving stability and efficacy of rhGH. Int. J. Pharm..

[35] Rejinold N.S., Jayakumar R., Kim Y.C. (2015). Radio frequency responsive nano-biomaterials for cancer therapy. J. Control. Release.

[36] Kim M.J., Park J.S., Lee S.J., Jang J., Park J.S., Back S.H., Bahn G., Park J.H., Kang Y.M., Kim S.H. (2015). Notch1 targeting siRNA delivery nanoparticles for rheumatoid arthritis therapy. J. Control. Release.

[37] Mitragotri S., Yoo J.-W. (2011). Designing Micro- and Nano-particles for Treating Rheumatoid Arthritis. Arch. Pharm. Res..

[38] Cassano D., Pocovi-Martinez S., Voliani V. (2018). Ultrasmall-in-Nano Approach: Enabling the Translation of Metal Nanomaterials to Clinics. Bioconjugate Chem..

[39] Torchilin V. (2011). Tumor delivery of macromolecular drugs based on the EPR effect. Adv. Drug Deliv. Rev..

[40] Mura S., Nicolas J., Couvreur P. (2013). Stimuli-responsive nanocarriers for drug delivery. Nat. Mater..

[41] Gouveia V.M., Lopes-de-Araujo J., Lima S.A.C., Nunes C., Reis S. (2018). Hyaluronic acid-conjugated pH-sensitive liposomes for targeted delivery of prednisolone on rheumatoid arthritis therapy. Nanomedicine.

[42] Alam M.M., Han H.S., Sung S., Kang J.H., Sa K.H., Al Faruque H., Hong J., Nam E.J., Kim I.S., Park J.H. (2017). Endogenous inspired biomineral-installed hyaluronan nanoparticles as pH-responsive carrier of methotrexate for rheumatoid arthritis. J. Control. Release.

[43] Ulmansky R., Turjeman K., Baru M., Katzavian G., Harel M., Sigal A., Naparstek Y., Barenholz Y. (2012). Glucocorticoids in nano-liposomes administered intravenously and subcutaneously to adjuvant arthritis rats are superior to the free drugs in suppressing arthritis and inflammatory cytokines. J. Control. Release.

[44] Wang S.P., Zhang J.M., Wang Y.T., Chen M.W. (2016). Hyaluronic acid-coated PEI-PLGA nanoparticles mediated co-delivery of doxorubicin and miR-542-3p for triple negative breast cancer therapy. Nanomedicine.

[45] De Castro Costa M., De Sutter P., Gybels J., Van Hees J. (1981). Adjuvant-induced arthritis in rats: a possible animal model of chronic pain. Pain.

[46] Nakamachi Y., Ohnuma K., Uto K., Noguchi Y., Saegusa J., Kawano S. (2016). MicroRNA-124 inhibits the progression of adjuvant-induced arthritis in rats. Ann. Rheum. Dis..

