# Supplementary material for: Hyaluronic Acid Coated Acid-Sensitive Nanoparticles for Targeted Therapy of Adjuvant-Induced Arthritis in Rats
Source: Molecules. 2019 Jan 2;24(1):146. doi: 10.3390/molecules24010146 (PMC6337373; doi:10.3390/molecules24010146)
Supplement: Supplementary file 1 [file molecules-24-00146-s001.pdf]

Hyaluronic Acid Coated Acid-Sensitive Nanoparticles for Targeted Therapy of Adjuvant-induced Arthritis in Rats

Changhui Yu [1]; Xiangyu Li [1]; Yufei Hou[#] [1]; Xiangxue Meng[#] [1]; Deli Wang; Jiaxin Liu [1]; Fengying Sun [1*] and Youxin Li [1*]

1 School of Life Sciences, Jilin University, Changchun, Jilin, 130012, China;

* Corresponding author: Fengying Sun and Youxin Li

# These authors contributed equally to this work

Email: sunfengying@jlu.edu.cn, liyouxin@jlu.edu.cn; Tel: +86-431-85155320; Fax: +86-431-85155320

Table S1 Compositions and characteristics of HAPNPs (n=3)

Batch	PCADK (mg)	egg PC (mg)	PEI (mg)	HA (μL)	Size (d.nm)	PDI	Zeta potential (mV)
F15	20	8	1	500	121.6 ±2.21	0.167 ±0.013	25.83 ±4.78

Note: Data are expressed as mean ± standard deviation.

Table S2 Compositions and characteristics of HANPs (n=3)

Batch	PLGA (mg)	egg PC (mg)	PEI (mg)	HA (μL)	Size (d.nm)	PDI	Zeta potential (mV)
F17	20	8	1	500	135.3 ±1.27	0.249 ±0.008	-15.20 ±1.40

Note: Data are expressed as mean ± standard deviation.

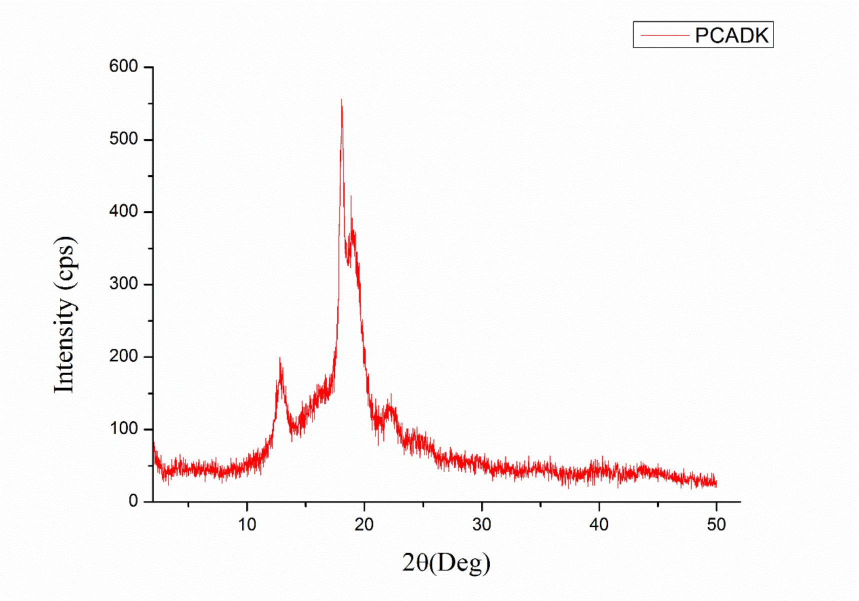

Figure S1 The XPRD sepctra of PCADK.

Powder X-ray diffraction data (XRD) were recorded on a Rigaku D/ SmartLab (3) X-ray diffractometer using a Cu target radiation source (50 kV, 200 mA). And the crystallinity of PCADK is 66.65%.
